# Supplementary material for: Waist-to-Height Ratio, Waist Circumference, and Body Mass Index in Relation to Full Cardiometabolic Risk in an Adult Population from Medellin, Colombia
Source: J Clin Med. 2025 Apr 1;14(7):2411. doi: 10.3390/jcm14072411 (PMC11989366; doi:10.3390/jcm14072411)
Supplement: Supplementary file 1 [file jcm-14-02411-s001.zip › jcm-3532076-supplementary.pdf]

Table S1. Definition of potential extreme/implausible values for anthropometric and biochemical variables	
	**Values**
**Glucose (mg/dl)**	<30 or >600
**Triglycerides (mg/dl)**	<10 or >1.000
**HDL-C (mg/dl)**	<10 or >118
**SBP (mm / Hg)**	<80 or >280
**DBP (mm/Hg)**	<50 or >195
**Waist (cm)**	<50 or >198
**BMI (Kg/m2)**	>70
SBP=Systolic Blood Pressure; DBP=Diastolic Blood Pressure; C=Cholesterol.	

Table S2. Identification of the study sample			
**Initial sample**	**Exclusion criteria**	***n* excluded**	**Remaining sample**
**69,883**	Missing values for anthropometric and biochemical variables	19,505	50,378
**50,378**	Potential extreme/implausible values for anthropometric and biochemical variables	2,589	47,789
**47,789**	Diagnostic of any of the following diseases: diabetes, kidney disease, cerebrovascular and cardiovascular disease	10,231	37,558
**37,558**	Missing values for covariates of age, sex, education level, ethnicity, area of residence, marital status, alcohol consumption, and smoking	8,322	**29,236 (Final sample)**
